# Risk Factors and Drug Resistance of the MDR Acinetobacter Baumannii in Pneumonia Patients in ICU

**DOI:** 10.1515/med-2019-0090

**Published:** 2019-10-25

**Authors:** Jichen Ren, Xiaomeng Li, Libo Wang, Mingzhu Liu, Ke Zheng, Yanrong Wang

**Affiliations:** 1 Jilin Tumor Hospital,Changchun 130012, China; 2 Endoscopy Center of China Japan Union Hospital to Jilin University, Changchun 130033, P.R.C, China

**Keywords:** ICU, MDR Acinetobacter baumannii, pneumonia, Drug resistance, Risk factors

## Abstract

**Objective:**

To investigate the risk factors and drug resistance of MDR Acinetobacter baumannii in pneumonia patients.

**Methods:**

From January 2013 to February 2016, 98 pneumonia patients with MDR Acinetobacter baumannii in our hospital ICU were selected as the observation group, and 49 pneumonia patients with not-MDR Acinetobacter baumannii in our hospital ICU were selected as the control group in accordance with the proportion of 2:1. Sputum samples were collected from the two groups for drug resistance, and the risk factors and prognosis of MDR Acinetobacter baumannii in pneumonia patients were given survey analysis.

**Results:**

The observation group was highly resistant to cefotaxime, piperacillin, imipenem, levofloxacin, gentamicin, tetracycline and ceftazidime, and was only sensitive to polymyxin. In addition to piperacillin, polymyxin B, the other antimicrobial drug resistance rates in the control group was significantly lower than in the observation group (P<0.05). Univariate analysis showed that diabetes, infection before hospitalization, admission 24h score of APACHE II and GCS scores, deep venous catheterization, and mechanical ventilation were related to the MDR Acinetobacter baumannii in pneumonia patients(P<0.05). Non conditional logistic regression analysis showed that diabetes mellitus, infection before hospitalization, admission 24h score of APACHE II and GCS scores were the independent risk factors for the MDR Acinetobacter baumannii in pneumonia patients(P<0.05).

**Conclusion:**

MDR Acinetobacter baumannii in pneumonia patients in ICU is common, where diabetes infection before hospitalization, admission 24h score of APACHE II and GCS scores are the main risk factors, and the vast majority of the antibiotics are resistant to the MDR Acinetobacter baumannii that can lead to poorer prognosis and followed-up of patients with increased mortality.

## 1 Introduction

Acinetobacter baumannii belongs to the genus of acreasia, is a gram-negative non-fermenting bacteria, in which only one or two antimicrobial agents are sensitive to Acinetobacter baumannii called pan resistant to Acinetobacter baumannii [1]. Pan-resistant Acinetobacter baumannii is widely distributed in water, dirt, soil and hospital environment. Especially, critically ill patients with respiratory disease has become an important pathogen [2,3]. Studies have revealed that pan-resistant Acinetobacter baumannii infection has obvious characteristics of the distribution of departments, including intensive care unit (Intensive Care Unit, ICU) infection rate near the top [4,5]. In particular, ICU patients are not only critically ill, but also they have suffered more invasive operation. And the vast majority of patients have used a large number of combined broad-spectrum antimicrobial agents, which makes their body immunity weaker and easier access to hospital infection. The current pan-resistant Acinetobacter baumannii can cause pulmonary infection, central nervous system infection, urinary tract infection, abdominal infection, blood infection. Among the most common is called pulmonary infection [6,7]. It has been found that the incidence rate of ICU pan-resistant Acinetobacter baumannii pneumonia is increasing year by year, and how to effectively control pneumonia caused by ICU-resistant Acinetobacter baumannii and reduce its incidence has attracted wide spread attention [8,9]. The resistance mechanism of the pan-resistant Acinetobacter baumannii mainly involves the production of β-lactamase, the loss of outer membrane porin, and the change of penicillin-binding protein (PBPs), etc. Besides, β-lactamase is considered to be the main cause of resistance to β-lactam drugs by P. albicans [10]. In this paper, we investigated the risk factors of acetobacter baumannii in patients with ICU pneumonia and analyzed the drug resistance.

## 2 Materials and methods

### 2.1 Research objects

Accepting criteria: 98 patients as an observation group was selected to stay in our hospital ICU ward after diagnosis of pan-resistant Acinetobacter baumannii pneumonia in January 2013 to February 2016, which is in line with hospital diagnostic criteria for pneumonia; lower respiratory tract secretions bacteria was checked for the pan-resistant Acinetobacter baumannii; excluding admission and ICU time less than 48h patients. At the same time in accordance with the ratio of 2:1 49 cases of Acinetobacter baumannii pneumonia as a control group was selected in our hospital ICU ward diagnosis and treatment of non-highly resistant patients with hospital-acquired pneumonia diagnostic criteria. According to to the United States ATS / IDSA hospital acquired pneumonia diagnosis and treatment guidelines, lower respiratory tract secretions of isolated pathogens ≥10^6^ cfu/ml and sputum quantitative culture separation pathogens ≥ 10^6^cfu / ml were collected by bronchoscopy or artificial airway, and qualified sputum for two consecutive isolates of the same pathogens was screened. Amino-glycosides antibiotics, pseudomonas cephalosporins, complex preparations containing beta-lactamase inhibitors, anti-Pseudomonas carbapenems antibiotics, fluoroquinolones antibiotics and other five kinds of antimicrobial drugs were used with at least one drug-resistant strain in each category, and only 1-2 kinds of antimicrobial agents (polymyxin, tigecycline) sensitive strains were used.

This study is in line with the medical ethics standards, and have access to the patient or family informed consent, and approved by the hospital ethics committee.

### 2.2 Drug resistance analysis

In the collection of sputum specimens, the use of natural sputum, bronchoscopy, artificial airway suction method, etc. with reference to the hospital infection prevention, control standard operating procedures to operate, daily collection of patients with sputum specimens for 2-3 days were carried out to ensure that sputum specimens qualified. We selected using phoenixTM-100 automatic bacterial identification and drug sensitivity meter produced by BD company in Japan, and the drug resistance analysis was carried out in our institute’s microbiology laboratory. The standard strain was treated with *Escherichia coli* (ATCC25922), *Pseudomonas aeruginosa* (ATCC27853) Quality control bacteria, the use of antimicrobial drugs, including cefotaxime, piperacillin, imipenem, polymyxin, gentamicin, levofloxacin, tetracycline, ceftazidime, drugs are from the Chinese pharmaceutical and biological products. The results were judged by reference to the standards set by the American Clinical Laboratory Standardization Committee (CLSI) to determine drug resistance, respectively. Antibacterial paper was produced by OXOID, UK.

### 2.3 Data survey

Basic information included name, sex, age, disease status, combined underlying disease, hospitalization time, laboratory examination data mainly for admission 24h admission APACHE II score, awareness assessment (GCS score), 7d after treatment APACHE II score and GCS score. Drug use included hormones, immunosuppressive agents or chemotherapy, invasive operations, including deep vein puncture, catheterization and time, mechanical ventilation, etc. Antibiotics use included cephalosporins, fluoroquinolones, polysaccharides, aminoglycosides, and follow-up prognosis followed by 6 months of death.

### 2.4 Statistical methods

The count data (X ± s) is expressed as a percentage, the *t* test (normal distribution) or rank sum test (non-normal distribution) is used for the comparison, and the number of measurement data is selected by the SPSS19.00 software. χ2 test and the univariate analysis of significant differences in the variables were used unconditionally with logistic regression analysis, with P <0.05 as a significant difference.

## 3 Results

### 3.1 Drug resistance comparison

After treatment, the observation group for cefotaxime, piperacillin, imipenem, gentamicin, levofloxacin, tetracycline, ceftazidime were highly resistant, and only polymyxin sensitive. In addition to piperacillin and polymyxin, the resistance rate of the control group to other antimicrobial agents was significantly lower than that of the observation group (P <0.05). See Table 1.

**Table 1:** Two groups of different antimicrobial resistance analysis (n)

Group	n	Cephalosporin Cefotaxine	Piperacillin Sieling	Imine Biapenem	Veterinary Science in China mycin	Levofloxacin Ofloxacin	Tetracyclic	Cephalosporin He pyridine	Polymyxa rhzomorph
Observation group	98	98	92	98	98	98	98	97	0
		(100.0%)	(93.9%)	(100.0%)	(100.0%)	(100.0%)	(100.0%)	(99.0%)	(0.0%)
Control group	49	41	42	39	37	31	35	30	0
		(83.7%)	(85.7%)	(80.0%)	(75.5%)	(63.3%)	(71.4%)	(61.2%)	(0.0%)
χ2		4.200	0.781	5.092	6.993	7.103	6.114	6.822	1.000
P		<0.05	>0.05	<0.05	<0.05	<0.05	<0.05	<0.05	>0.05

### 3.2 Univariate analysis of risk factors

We compared the clinical data of all patients with independent variables as a single factor analysis. The results showed that diabetes mellitus, pre-infection hospital stay, admission 24h APACHE II score, GCS score, deep vein catheterization, mechanical ventilation and other factors and ICU Mycobacterium pneumoniae are significantly correlated (P <0.05). See Table 2.

**Table 2:** Univariate analysis of pan-resistant Acinetobacter baumannii infection in ICU pneumonia patients

Virable	Observation group(n=98)	Control group(n=49)	t or χ2	P
Diabetes	24(24.5%)	4(8.2%)	4.022	<0.05
Hospitalization time before (d)	7.66±1.34	4.22±1.42	5.109	<0.05
APACHE II grade	24.99±2.44	15.33±3.51	4.445	<0.05
GCS grade	11.66±2.15	8.92±1.55	5.091	<0.05
Deep vein catheterization	43(43.9%)	9(18.4%)	3.984	<0.05
Mechanical ventilation	52(53.1%)	11(22.4%)	4.771	<0.05

### 3.3 Multivariate analysis of risk factors

We analyzed the unconvolable logistic regression analysis of the seven variables with significant differences in univariate analysis. The results showed that diabetes mellitus, hospital stay before admission, APACHE II score within 24 hours of admission, GCS score, resulted in pan-resistant Acinetobacter baumannii infection with independent risk factors (P <0.05) shown in table 3.

**Table 3:** Multivariate analysis of Pan common resistance to Acinetobacter baumannii infection in ICU pneumonia patients

Variable	P value	ORvalue	OR(95%CI)
Diabetes	0.000	5.982	2.871-14.598
Hospitalization time before infection	0.003	5.624	1.893-17.492
APACHE II grade	0.012	5.645	1.513-30.104
GCS grade	0.003	6.559	1.689-13.922

### 3.4 Prognosis comparison

The APACHE II score and GCS score of the observation group were significantly lower than those of the control group (P <0.05). The mortality rate of the observation group was also lower than that of the control group (P <0.05). See Table 4.

**Table 4:** Comparison of prognosis in both groups

Group	n	APACHE IIgrade	GCSgrade	death rate
Observation group	98	12.49±2.44	7.29±1.44	19(19.4%)
Control group	49	8.13±1.94	5.09±1.33	3(6.1%)
χ2		5.298	4.885	3.921
P		<0.05	<0.05	<0.05

## 4 Discussions

Acinetobacter baumannii is a simple conditional pathogen, which is widely found in nature and has a strong ability to adapt to the environment and the ability to quickly obtain resistance to become an important pathogen of hospital infection [10]. Studies have shown that when first non-fermentative bacteria are isolated, the number of resistant strains are high and most of them pan-resistant strains. Because of its lack of specific clinical manifestations, pan-resistant Acinetobacter baumannii pneumonia has similar symptoms to other types of pneumonia, therefore the early diagnosis rate is relatively low, so the treatment effect is poor with high mortality [11,12]. At the same time ICU patients have low immunity, poor or restricted respiratory function, generally low defence capacity, and are elderly, so that basic disease is more serious, more invasive making it a high incidence of hospital infection [13,14]. This study showed that APACHE II score and GCS score were significantly lower in the observation group than in the control group (P <0.05), and the mortality rate in the observation group was also lower than that in the control group (P <0.05) Acinetobacter baumannii pneumonia can lead to a worse prognosis in patients with increased mortality.

Acinetobacter baumannii with natural resistance is treated with a variety of antimicrobial drugs, making the pan-resistant strains increase year by year [15]_，_especially pan-resistant Acinetobacter baumannii on penicillins, sulfonamides, aminoglycosides, cephalosporins class, carbapenems and other antimicrobial drugs. This study shows that the observation group is highly resistant to cefotaxime, piperacillin, imipenem, gentamicin, levofloxacin, tetracycline and ceftazidime, and is only sensitive to polymyxin. In addition to piperacillin and polymyxin, the resistance rate of the control group to other antimicrobial agents was significantly lower than that of the observation group (P <0.05). From the mechanism analysis, β-lactamase is the main mechanism that is desirable for resistancing β-lactamase antibiotics, acetinase acts on the β-lactam ring, making β- Lactam bond cleavage and loss of antibacterial activity [16]. Polymyxin has a good antibacterial activity against pan-resistant Acinetobacter baumannii, especially for severe pneumonia infection, but with more adverse reactions. To this end for the pan-resistant Acinetobacter baumannii infection in patients with pneumonia in the drug sensitivity results did not come out before the empirical selection of antimicrobial drugs, drug susceptibility should be decisive after the drug withdrawal, according to the results of drug susceptibility to choose antibiotics [17].

The panoramic resistance to Acinetobacter baumannii can be of adherent or colonized form and exists in different tissues and systems of the human body and organs In patients with low immunity, Acinetobacter baumannii can proliferate and produce pneumonia [18,19]. In the process of deep vein catheterization and mechanical ventilation, medical instruments will destroy the normal protective mucosa of patients’ respiratory tract, the conventional air pressure and hemodynamics are prone to change, thus increasing the risk of pan-resistant Acinetobacter baumannii infection [20]. In this hospital, ICU patients with anti-Pneumocystis aeruginosa pneumonia are admitted with most respiratory diseases. The patients usually have poor expectoration, are on a variety of antibiotics, have respiratory (anatomical or physiological) functional damage, and be on mechanical ventilation among other factors. In this study, univariate analysis showed that diabetes mellitus, hospital stay before admission, APACHE II score, GCS score, deep venous catheterization, mechanical ventilation and other factors were significantly associated with ICU endemic resistant Acinetobacter baumannii pneumonia (P <0.05 ). Univariate logistic regression analysis showed that diabetes mellitus, pre-infection hospital stay, APACHE II score within 24 hours after admission, GCS score led to the independent risk factors of pan-resistant Acinetobacter baumannii infection (P <0.05). APACHE II score and GCS score is a system that can reflect the seriousness of disease and the ability of disease resistance reserve. It can be used to evaluate the prognosis of patients. It is widely used in ICU department. The higher the APACHE II score and the GCS score, the more serious the disease is and the moreopportunities for the appearance drug-resistant strains [21,22]. Deep venous catheterization and mechanical ventilation destroys the normal respiratory barrier, causing airway mucosal injury, and thus affects normal airway pressure and physiological hemodynamics, which increases lung infection opportunities [23]. Diabetes can destroy the control relationship between bacteria, leading to the destruction of human micro-ecological balance, which is conducive to the formation of pan-resistant species of Acinetobacter baumannii. The longer the hospital stay before infection, but also increased the risk of hospital infection in patients with the disease, can lead to the advantages of pan-resistant Acinetobacter baumannii growth [8,10].In general, ICU pneumonia in patients with pan-resistant Acinetobacter baumannii infection is more common, with diabetes, pre-infection hospital stay, admission 24h APACHE II score, GCS score as the main risk factors. Pan-resistant Acinetobacter baumannii is the most resistant strain to antimicrobial drugs, and infection leads to poor prognosis in patients with follow-up mortality increased.
